# Enhancing Physiological Complexity Through Mindfulness: A Wearable-Based Intervention for First Responders and Their Partners

**DOI:** 10.3390/healthcare14111532

**Published:** 2026-06-01

**Authors:** Thurmon E. Lockhart, Rahul Soangra, Christopher Frames, Sun-Kyung Lee, Amberlee Martin, Susanne Lee, Abigail H. Gewirtz

**Affiliations:** 1 School of Biological and Health Systems Engineering, Arizona State University, Tempe, AZ 85281, USA; thurmon.lockhart@asu.edu (T.E.L.); cframes@asu.edu (C.F.); 2 Crean College of Health and Behavioral Sciences, Chapman University, Orange, CA 92866, USA; soangra@chapman.edu; 3 Fowler School of Engineering, Chapman University, Orange, CA 92866, USA; 4 T. Denny Sanford Harmony Institute, Arizona State University, Phoenix, AZ 85281, USA; sunkyung.lee@asu.edu; 5 Reach Institute, Department of Psychology, College of Liberal Arts and Sciences, Arizona State University, Tempe, AZ 85281, USA; amberlee.martin@asu.edu (A.M.); susanne.lee@asu.edu (S.L.); 6 School of Social Work, Hebrew University of Jerusalem, Jerusalem 9190501, Israel

**Keywords:** heart rate variability, HRV complexity, mindfulness, first responders, wearable technology, autonomic regulation, sample entropy, multiscale entropy, ecological momentary assessment, stress management

## Abstract

**Highlights:**

**What are the main findings?**

- A one-month wearable-based mindfulness intervention significantly increased HRV complexity metrics (Sample Entropy and Multiscale Entropy at lower scales) among first responders, indicating improved autonomic regulation.
- Reductions in Recurrence Rate and Determinism post-intervention suggest enhanced cardiovascular adaptability and reduced physiological rigidity, while traditional time-domain metrics (Mean HR, SDNN, RMSSD) remained unchanged.

**What are the implications of the main findings?**

- Nonlinear HRV metrics are more sensitive than traditional measures for detecting autonomic changes from digital mindfulness interventions, supporting their use in evaluating stress management programs in high-risk occupational populations.
- Wearable-based mindfulness strategies show promise for integration into occupational health programs for first responders; future research should examine longitudinal effects and mechanisms mediating these autonomic adaptations.

**Abstract:**

**Background/Objectives:** First responders frequently encounter high-stress environments that challenge physiological resilience and autonomic regulation. Heart rate variability (HRV) complexity is a critical marker of adaptive capacity and stress regulation. This study assessed the impact of a wearable-based mindfulness intervention on HRV complexity among first responders using a smartwatch. **Methods:** A total of 87 first responders participated in a one-month wearable-based intervention. Participants wore Garmin Vivosmart 5 devices to continuously collect PPG data (photoplethysmogram), focusing on beat-to-beat intervals (BBIs). The intervention involved daily Ecological Momentary Assessments (EMAs) and individual randomization to either a mindfulness message, a prompt to access an audio exercise, or no-treatment/control; interventions were delivered via the MYAPT.MIND mobile application. HRV metrics, including Sample Entropy, Multiscale Entropy (MSE), Recurrence Rate (RR), and Determinism (Det), were analyzed pre- and post-intervention/control using paired-samples *t*-tests. **Results:** Significant improvements were observed in HRV complexity metrics post-intervention. Sample Entropy increased (M = 1.42, SD = 0.11) compared to pre-intervention (M = 1.39, SD = 0.10; *p* = 0.007). MSE also showed significant gains (*p* = 0.038), particularly at lower scales, indicating enhanced short-term autonomic flexibility. Reductions were noted in RR (*p* = 0.025) and Det (*p* = 0.018), suggesting improved cardiovascular adaptability and reduced physiological rigidity. Other traditional time-domain metrics, such as Mean HR, SDNN, and RMSSD, did not exhibit significant changes. **Conclusions:** The wearable-based intervention significantly enhanced HRV complexity, reflecting improved autonomic regulation and adaptive capacity in first responders. These findings support the integration of digital mindfulness strategies for stress management in high-risk occupations. Future research should explore the longitudinal effects and mechanisms mediating these autonomic adaptations.

## 1. Introduction

Smart wearable devices have revolutionized health monitoring by enabling continuous, long-term data collection that surpasses traditional short-term methods. These devices offer real-time insights into various health metrics, such as exercise patterns, sleep quality, and physiological responses, facilitating a comprehensive analysis of individual health trends [1]. In particular, the utilization of smartwatches for heart rate variability (HRV) analysis has significantly advanced the monitoring of stress levels, especially among high-risk groups like emergency response workers [2]. By providing immediate feedback on physiological states, these devices support a deeper understanding of personal health dynamics and enable timely, tailored interventions to manage stress effectively [3].

Recent advancements in wearable technology, particularly the integration of accelerometers and photoplethysmography (PPG) sensors, have enhanced the capability for continuous health monitoring over extended periods. Such advancements facilitate the monitoring of diverse physiological and activity-related metrics, offering a richer understanding of health patterns. Previous research has demonstrated that individuals with chronic conditions, such as stroke, exhibit distinct activity and health patterns that can be effectively tracked using wearable devices [4]. The ability to capture daily activities and physiological metrics over multiple days provides a wealth of data for understanding health trends and identifying potential anomalies [5,6].

Smartwatches equipped with PPG sensors allow for continuous HRV analysis, a critical indicator of autonomic nervous system (ANS) activity. HRV reflects the dynamic balance between the sympathetic and parasympathetic branches of the ANS, which regulate stress responses. A higher HRV is typically associated with relaxation and resilience, while lower HRV indicates heightened stress levels. However, scalar HRV indices such as SDNN and RMSSD capture only the magnitude of beat-to-beat variability and may fail to detect the structural degradation of autonomic dynamics that accumulates under chronic occupational stress. Nonlinear complexity metrics including Sample Entropy, Multiscale Entropy, and recurrence quantification measures provide a more sensitive characterization of the multi-scale regulatory capacity of the autonomic nervous system, reflecting the system’s ability to flexibly adapt rather than merely its average level of variability. Modern smartwatches from brands like Apple and Garmin leverage advanced optical sensors and algorithms to continuously monitor HRV, offering real-time insights into stress levels and enabling personalized interventions such as guided breathing exercises and mindfulness prompts. This capability is particularly beneficial for emergency responders, who routinely operate in high-stress environments. Wearables may foster self-awareness and promote healthier coping strategies, empowering these individuals to manage stress proactively. Moreover, HRV data can facilitate the assessment of stress reduction techniques, contributing to a balanced and stress-resilient lifestyle. Continuous monitoring also supports the early identification of health anomalies, such as fluctuations in heart rate and activity levels, which may signal underlying health conditions [7].

Physiological complexity, characterized by the dynamic interplay between stability and adaptability within biological systems, plays a critical role in maintaining health and resilience [8]. The concept of “optimal variability” in heart rate dynamics underscores the intricate regulatory processes governed by multiple control systems and feedback loops that sustain healthy physiological functioning. This balance reflects a state where the system remains adaptable, neither overly predictable nor excessively random [9]. Measures of HRV complexity, such as entropy and fractal scaling, offer valuable insights into the body’s capacity for stress regulation. A decline in HRV complexity can indicate compromised autonomic regulation, as seen in conditions associated with chronic stress, fatigue, and cardiovascular issues. Critically, first responders including firefighters, paramedics, and law enforcement officers are chronically exposed to unpredictable, high-intensity stressors that progressively erode autonomic complexity, predisposing them to burnout, post-traumatic stress disorder, and elevated cardiovascular risk [10,11,12,13,14,15]. This population therefore represents an important target for complexity-sensitive physiological monitoring and intervention. Conventional time-domain HRV indices, including SDNN and RMSSD, quantify the overall magnitude of beat-to-beat variability but are insensitive to the temporal structure and multi-scale dynamics of autonomic regulation; the properties most vulnerable to chronic stress exposure [16]. Sample Entropy was selected as a metric of signal irregularity and unpredictability, reflecting the autonomic system’s capacity to generate non-repetitive, adaptive beat-to-beat patterns [17,18]. Multiscale Entropy extends this assessment across multiple physiological time scales, capturing regulatory complexity at both short-and long ranges. Recurrence Rate and Determinism, derived from Recurrence Quantification Analysis, quantify the proportion of deterministic, repetitive structure in heart rate dynamics; under chronic stress, both metrics increase, reflecting pathological rigidity and reduced cardiovascular adaptability [19]. These four metrics were selected because they have demonstrated greater sensitivity to autonomic dysregulation compared to conventional indices in high-stress tactical occupational populations. By monitoring HRV complexity through wearable devices, we can assess the cumulative impact of stress and evaluate the effectiveness of interventions aimed at restoring physiological balance. Integrating HRV complexity analysis into wearable health monitoring holds significant potential for enhancing the well-being of emergency responders exposed to unpredictable and demanding environments.

To date, there is limited evidence evaluating whether wearable-integrated mindfulness interventions can modulate physiological markers of stress. Prior studies have examined mindfulness interventions delivered via mobile applications [20,21] and, separately, the use of wearable devices for HRV [22,23], but few studies integrated these components into a unified intervention framework. Existing digital mindfulness studies also rely on self-reported outcomes [21,24], and physiological investigations are typically conducted in controlled conditions or short-duration settings [22,25]. Addressing these gaps, the present study is novel in integrating wearable-based physiological monitoring with digital mindfulness intervention delivery in a real-world setting.

Moreover, despite the established sensitivity of nonlinear HRV metrics to autonomic dysregulation in high-stress populations, no prior study has examined whether a wearable-delivered mindfulness intervention can restore HRV complexity in active first responders over a one-month free-living period. In this study, we assessed HRV in 87 emergency responders over a one-month period to examine the preliminary impact of a wearable-based intervention on HRV complexity and related physiological metrics. Baseline HRV complexity values, measured over two days at baseline, were compared to HRV complexity values obtained during a two-day assessment after one month to evaluate changes in HRV. The primary objective was to determine whether the wearable-based intervention could modulate key indicators of autonomic nervous system function, with a particular focus on HRV complexity measures such as entropy and fractal scaling. This investigation aims to deepen our understanding of how digital health tools can enhance physiological resilience and stress regulation among emergency response workers, who face continuous exposure to high-stress environments.

## 2. Materials and Methods

### 2.1. Participants

All methods were performed in accordance with the guidelines of the Declaration of Helsinki and approved by the Institutional Review Board of Arizona State University (STUDY00017540), and all participants provided informed consent. Participants were recruited to participate in a wearable-based stress management program using mobile technology and wrist-based wearable devices. Eligibility criteria included (a) being employed as a first responder (i.e., emergency medical technician, firefighter, or police officer) in two southern states in the U.S. (Tennessee and Kentucky), and (b) having at least one child between the ages of 4 and 13 years. Spouses or partners of consented first responders were invited to participate in the study. A total of 100 individuals participated in the study; however, due to HRV data quality issues (n = 12) and health-related issues not related to the study (n = 1), the final analysis included 87 participants comprising first responders (n = 70) and their spouses (n = 17, two of whom also worked as first responders).

Participants’ mean age was 38.7 years (SD = 6.4, range = 27–59) and 66.7% (n = 58) were male. The sample was predominantly White (89.7%), with 6.9% Black/African American and 3.4% other races. A third of the sample had a high school degree, 23.0% an associate’s degree, 33.3% a bachelor’s degree, and 10.3% a graduate degree. Most participants worked full-time or more (88.5%), with 5.7% part-time workers and 5.7% homemakers. Participants identified as firefighter (41.4%), paramedic (23.0%), emergency medical technician (20.7%), police officer (19.5%), or other (6.9%; e.g., dispatcher, emergency manager). Annual household income was categorized as less than $50,000 (18.4%), $50,000–$74,999 (18.4%), $75,000–$99,999 (33.3%), $100,000–$149,999 (16.1%), or $150,000 or more (13.8%). Participants reported being married (82.8%), divorced (6.9%), never married (5.7%), widowed (3.4%), or separated (1.1%).

### 2.2. Procedures

**Figure 1:**
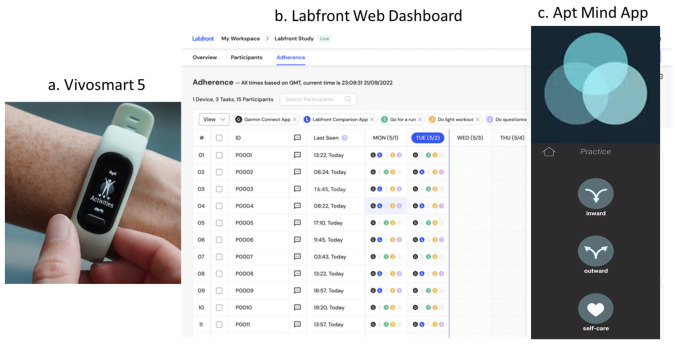
Wearable and digital tools used in the study. (**a**) Garmin Vivosmart 5 smartwatch for PPG data collection; (**b**) LabFront web dashboard for data retrieval from cloud; (**c**) MYAPT.MIND application for intervention delivery and subjective questionnaires.

First responders and their spouses or partners were recruited via multiple strategies including email lists of Kentucky and Tennessee first responders, social media advertisements (e.g., Facebook), word-of-mouth, refer-a-friend referrals, and printed flyers. Once a participant provided consent, staff scheduled a virtual welcome session and sent each participant a survey link for baseline demographic information. A packet containing printed materials and a Garmin Vivosmart 5 device (Figure 1a) was mailed to each participant prior to the scheduled session. The Garmin Vivosmart 5 utilizes Garmin’s Elevate v4 multi-wavelength PPG sensor to capture continuous beat-to-beat intervals. Prior validation studies have demonstrated acceptable agreement between PPG-derived and ECG-derived HRV metrics under resting and low-to-moderate activity conditions, supporting the use of such devices for ambulatory HRV monitoring in free-living environments. Although PPG signals are more susceptible to motion artifacts compared to gold-standard ECG particularly during vigorous activity the 24-h continuous monitoring protocol in this study encompasses substantial periods of sedentary and low-intensity behavior, during which PPG signal quality is optimal.

The selection of a consumer-grade wearable was intentional, aiming to enhance ecological validity, promote participant adherence over the one-month monitoring period, and reflect real-world conditions relevant to wearable-based occupational health interventions. Additionally, the LabFront platform implemented automated artifact detection and correction procedures prior to HRV computation, further reducing the impact of motion-related noise on the derived metrics [26].

During the welcome session, participants were instructed to wear the smartwatch for two days prior to the study to collect baseline beat-to-beat interval (BBI) data. Independent validation studies have demonstrated that PPG-derived HRV indices including time-domain metrics and nonlinear entropy measures show strong agreement with ECG-derived counterparts under resting and low-to-moderate activity conditions, with mean absolute errors typically within acceptable clinical thresholds [27,28,29]. The study utilized a LabFront database (Figure 1b) to extract data from the Garmin sensors objectively. Participants also provided projected times for wake, sleep, work start, and work end for the 30-day intervention period. Following baseline data collection, participants received two daily surveys (i.e., ecological momentary assessments; EMA) and intervention activities from the study application, named MYAPT.MIND (Figure 1c), for 30 days while BBI data continued to be collected (additional information about the EMA and intervention activities can be found in [30]).

The first daily survey (EMA 1) was sent randomly between two hours after the projected wake-up time and four hours before the projected sleep time. Participants had 10 min to complete EMA 1, with a reminder sent after 5 min if needed. Upon completion or timeout of EMA 1, participants were randomized each day into one of three intervention conditions: (a) a notification to listen to a 2–5 min audio-based mindfulness exercise via the MYAPT.MIND app (e.g., a body scan, kindness meditation); (b) a pop-up mindfulness message (e.g., “think of someone you appreciate,” “take 10 deep breaths”); or (c) no-notification control. On average, each participant was assigned to the app/audio mindfulness exercises one third of the time (10 days), to the brief message condition one third of the time (10 days) and to the control (no intervention condition) one third of the time/10 days. Participants had one hour to complete the assigned activity. Following the hour, the second EMA (EMA 2) was sent, requiring completion within 60 min, with a reminder sent after 30 min if necessary. This process repeated daily until the 30-day period was completed. At the end of the 30 days, participants attended a virtual exit session with research staff and returned the Garmin Vivosmart 5 devices for reuse in future studies.

Recording Period and Wear Protocol. BBI data were captured continuously across the full 24-h day for each participant, encompassing both waking and sleeping hours. This approach was adopted to obtain comprehensive, ecologically representative HRV indices that reflect the total autonomic burden experienced across a participant’s daily and nocturnal cycle, rather than any single physiological state. Participants were instructed to wear the Garmin Vivosmart 5 device at all times, with removal permitted only for device charging and in situations involving prolonged water exposure (e.g., bathing, showering, or swimming). Segmentation of data into daytime or nighttime epochs was not applied; all available valid BBI data within each 24-h recording period were used for HRV metric computation.

Minimum Wear Criteria and Data Quality. To ensure that HRV estimates were based on sufficient and reliable data, a minimum wear criterion was applied: participants were required to have at least 18 h of valid BBI data per 24-h analysis day. Participants who did not meet the minimum data quality threshold on one or both analysis days were excluded from the final dataset (n = 12 excluded due to HRV data quality issues, as noted in Section 2.1).

Summary of Baseline and Post-Intervention Windows. The two-day baseline period corresponded to the two days immediately preceding the start of the 30-day intervention. The two-day post-intervention period corresponded to the two days after the 30-day monitoring window. For each participant, HRV metrics were computed separately for each qualifying day within both assessment windows using the full 24-h BBI recording for that day. The resulting per-day HRV values were then averaged across the two days to yield a single pre-intervention score and a single post-intervention score per participant for each metric. This day-averaging approach reduces the influence of day-to-day biological variability and produces stable, representative physiological estimates for each assessment period. Paired-samples *t*-tests were applied to the resulting participant-level pre- and post-intervention averages.

### 2.3. HRV Data Analysis

The Garmin Vivosmart 5 captures PPG signals at a sampling frequency of 25 Hz using Garmin’s Elevate v4 multi-wavelength optical sensor, from which beat-to-beat intervals were extracted using the LabFront platform’s proprietary peak detection algorithm applied to the raw PPG waveform. To ensure signal quality, an automated artifact correction protocol was applied within LabFront prior to HRV metric computation. Beat-to-beat intervals falling outside the physiologically plausible range of 300–2000 ms, or deviating more than 20% from the local median of the surrounding five beats, were flagged and excluded from analysis. No interpolation was applied to replace rejected intervals; instead, affected segments were removed entirely to preserve the integrity of the nonlinear HRV metrics, which are sensitive to artificial regularity introduced by interpolated values. A paired-samples t-test was conducted to evaluate the impact of the mindfulness stress management program on participants’ HRV metrics. All statistical analyses were performed using JMP Pro 18.0.2 (SAS Institute Inc., Cary, NC, USA). HRV metrics examined included traditional time-domain measures (Mean HR, SDNN, RMSSD, Stress Index [SI], SNS activity, Parasympathetic Nervous System [PNS] activity) and nonlinear complexity measures (Sample Entropy, Multiscale Entropy [MSE], Recurrence Rate [RR], and Determinism [Det]).

## 3. Results

Across the 30-day intervention period, participants engaged in mindfulness activities on an average of 11.3 days (56.5% of intervention days), or 37.7% of total study days (including control days when no intervention notification was delivered). Of note, participants reported engaging in mindfulness activities on an average of 1.8 days during control periods, suggesting some self-initiated mindfulness practice. Adherence was calculated based on the proportion of completed activities relative to the number of notifications delivered for each intervention condition. Participants completed 43.7% of the MYAPT.MIND audio exercises (M = 4.5 of 10.3 prompted days) and 51.3% of the brief mindfulness exercises (M = 5.0 of 9.6 prompted days). Across all intervention prompts, overall adherence was 47.3%, indicating moderate compliance.

The paired-samples t-test results indicated statistically significant increases in several nonlinear HRV parameters from pre- to post-intervention.

Across the sample, there was a significant increase in Sample Entropy from pre-intervention (M = 1.39, SD = 0.10) to post-intervention (M = 1.42, SD = 0.11), t(86) = 2.78, *p* = 0.007, d = 0.30, indicating improved complexity and reduced regularity in HRV patterns. Multiscale Entropy (MSE) also showed a significant increase from pre-intervention (M = 1.67, SD = 0.11) to post-intervention (M = 1.69, SD = 0.13), t(86) = 2.11, *p* = 0.038, d = 0.23, suggesting increased adaptability of the autonomic nervous system.

Additionally, Recurrence Rate (RR) decreased from pre-intervention (M = 29.21, SD = 17.23) to post-intervention (M = 28.95, SD = 12.20), t(86) = −2.30, *p* = 0.025, d = −0.25, indicating less repetitive heart rate patterns. Determinism (Det) also decreased slightly from pre-intervention (M = 97.67, SD = 30.20) to post-intervention (M = 97.59, SD = 24.23), t(86) = −2.41, *p* = 0.018, d = −0.26, reflecting reduced predictability in heart rate dynamics.

However, no statistically significant differences were found for Mean HR, SDNN, RMSSD, Stress Index (SI), SNS activity, or PNS activity (*p* > 0.05). These results suggest that the mindfulness intervention influenced complex and nonlinear aspects of heart rate variability, reflecting improved autonomic regulation and stress resilience in participants. Full descriptive statistics and t-test results for all HRV parameters are presented in Table 1.

### 3.1. Sample Entropy Analysis

**Figure 2:**
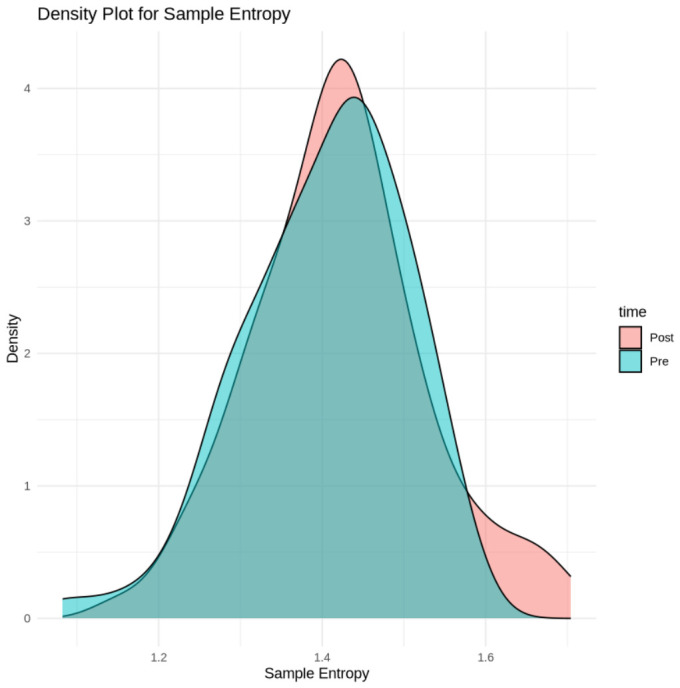
Density plots showing distribution of pre- and post-intervention Sample Entropy values. A noticeable shift toward higher entropy values is observed post-intervention.

**Figure 3:**
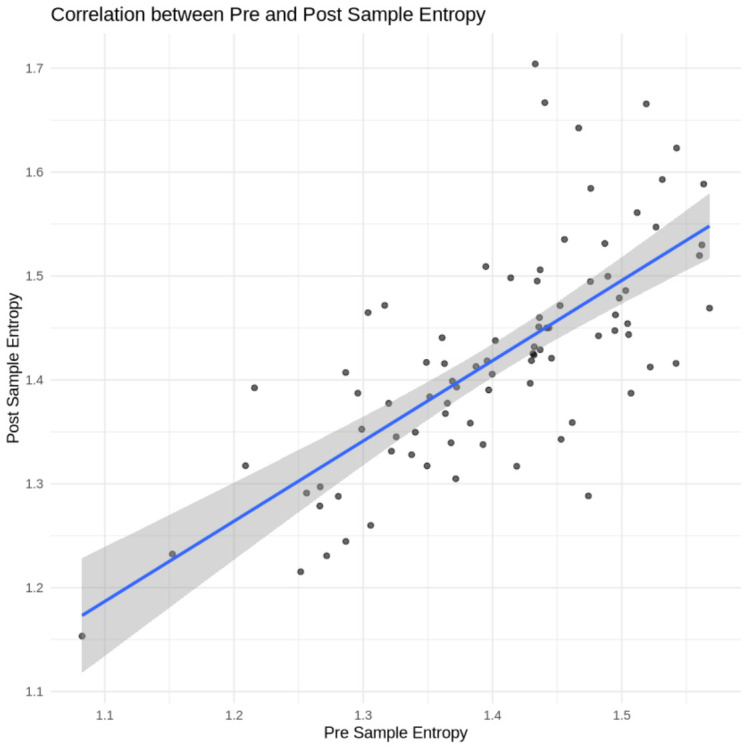
Correlation plot showing the linear relationship between pre- and post-intervention Sample Entropy values, illustrating individual consistency in adaptive responses. The blue line represents the linear regression fit, and the grey shaded band denotes the 95% confidence interval of the regression estimate.

Density and correlation plots between pre- and post-intervention Sample Entropy indicate a relatively smooth distribution and a linear relationship (Figure 2). The paired *t*-test on Sample Entropy resulted in a statistically significant change (*p* < 0.05). This suggests that the intervention between the pre- and post-measurements may have affected the regularity or predictability of the heart rate signals. The correlation plot shows a clear linear trend, suggesting that participants with higher pre-intervention entropy tend to have higher post-intervention entropy (Figure 3). This indicates consistency in individual differences—individuals generally maintain their relative position even if there is an overall shift.

### 3.2. Multiscale Entropy Analysis

**Figure 4:**
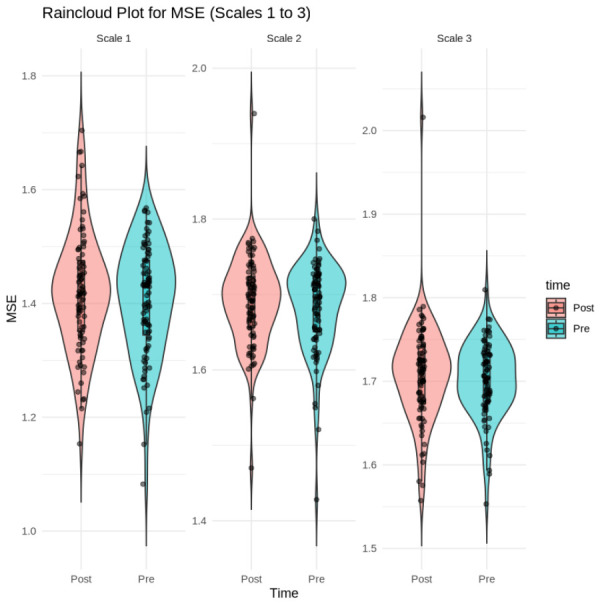
Raincloud plots for MSE scales 1 to 3, illustrating the distribution, central tendency, and individual data points for pre- and post-intervention values.

The raincloud plots for MSE scales 1–3 reveal the overall spread and central tendency for these scales (Figure 4). While the *t*-tests for MSE Scale 1 and Scale 2 showed significance, results for Scale 3 and beyond were not statistically significant. This indicates that certain scales of complexity in HRV may be more sensitive to the applied intervention than others.

**Figure 5:**
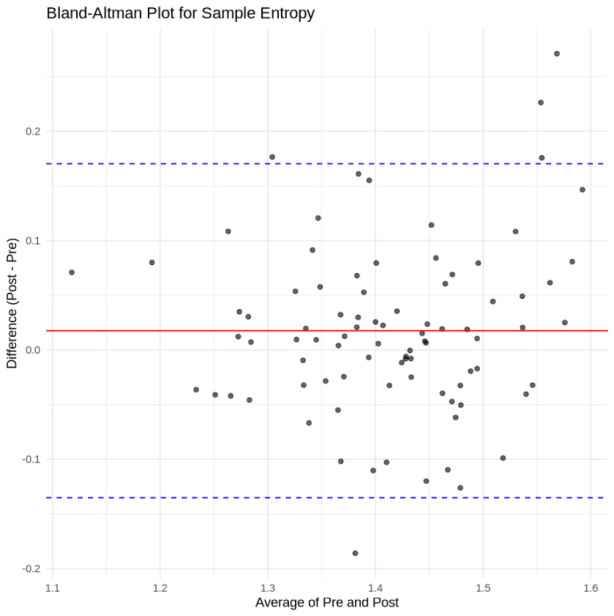
Bland–Altman plot for Sample Entropy showing agreement between pre- and post-intervention measurements. Most data points lie within the 95% limits of agreement (dotted lines), with a systematic mean (red line) shift indicating a consistent intervention effect.

The Bland–Altman plot for Sample Entropy (Figure 5) helps visualize the agreement between pre- and post-measurements by showing the mean difference and the limits of agreement. The placement of most data points within the 95% limits suggests that the differences are consistent, though the significant shift in mean difference (highlighted by the red line) reinforces the presence of a systematic change.

### 3.3. Recurrence Rate and Determinism Analysis

**Figure 6:**
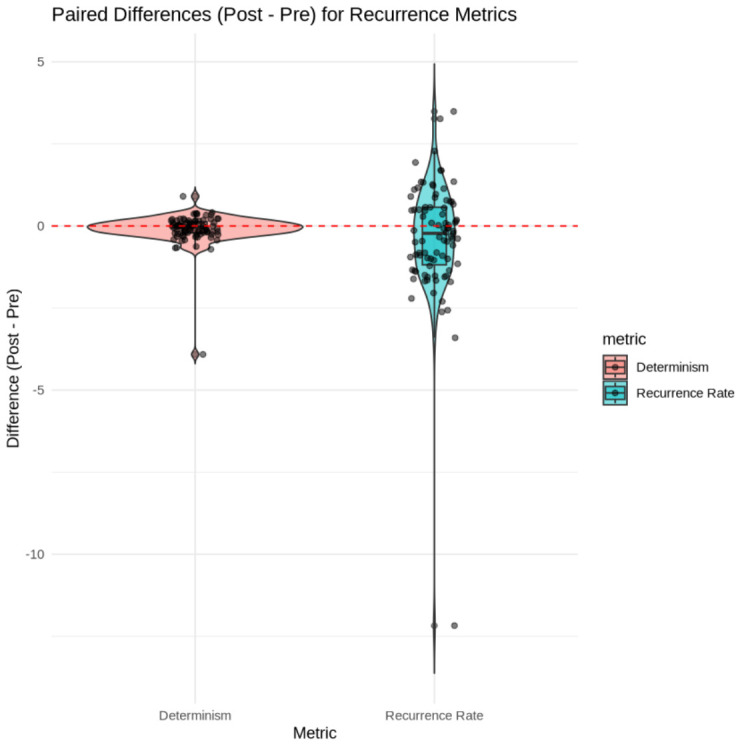
Paired differences (post–pre) for Recurrence Rate and Determinism, illustrating reductions in both metrics post-intervention and indicating enhanced cardiovascular adaptability and reduced physiological rigidity.

The paired differences plot for Recurrence Rate and Determinism (Figure 6) shows the distribution of changes between pre- and post-intervention. These metrics relate to the repetitiveness and predictability in the time series and indicate how the overall structure of heart rhythm has shifted post-intervention.

## 4. Discussion

The present study investigated the effects of a wearable-based stress management program on HRV metrics among first responders, emphasizing parameters rooted in nonlinear dynamics. While the examination of the differential effects of the three conditions (i.e., daily within-individual randomization to no-intervention, mindful message, or the MYAPT.MIND app) is beyond the scope of the current investigation, it will be the focus of future studies. This study examined the overall impact of the intervention on HRV metrics.

Before interpreting the findings of this study, several important limitations must be acknowledged. This investigation was designed as a preliminary, single-group pre-post pilot study with only within-subject randomization to interventions or a control condition, which precludes causal inference and leaves observed changes susceptible to time-dependent confounding factors such as seasonal variation, occupational stress cycles, or regression to the mean. The sample size was modest, and participants were drawn from a self-selected volunteer pool of first responders at a single agency, limiting statistical power and the generalizability of findings to broader first-responder populations or other high-stress occupational groups. HRV data were acquired using a consumer-grade photoplethysmography-based wearable device rather than gold-standard electrocardiography, which introduces potential measurement error attributable to motion artifact and optical signal noise, particularly during high-activity periods. Device wear was participant-directed, and brief removal periods for charging or water exposure may have introduced intermittent data gaps that, despite quality screening, could have influenced 24-h HRV estimates. The absence of concurrent ECG validation is acknowledged as a limitation; however, independent validation studies of Garmin PPG-derived HRV indices against simultaneous ECG have reported acceptable agreement for time-domain and nonlinear complexity metrics under resting and low-to-moderate activity conditions [27]. Additionally, several physiological and behavioral confounders known to influence HRV including sleep quality and duration, physical activity intensity, caffeine intake, and shift schedule variability were not systematically measured or controlled in this study. Given the heterogeneous and unpredictable occupational schedules of first responders, experimental control of these variables was not feasible in a free-living deployment; however, their uncontrolled influence represents a meaningful constraint on causal interpretation of the observed HRV complexity changes. The use of 24-h continuous recording and two-day averaging at each assessment window partially mitigates the impact of acute single-day fluctuations, but does not eliminate the potential for systematic confounding across the intervention period. Finally, multiple HRV outcomes were examined using univariate paired *t*-tests; although Bonferroni correction was not applied given the intercorrelated nature of the metrics and the exploratory study design, the possibility of false-positive findings cannot be excluded. Collectively, these constraints require that all results presented herein be interpreted as hypothesis-generating observations warranting replication in larger, adequately powered, randomized controlled trials before any clinical or operational conclusions can be drawn.

The concept of “optimal variability” in heart rate dynamics underscores the balance between stability and adaptability within physiological systems. This balance reflects the intricate interactions among multiple control mechanisms, feedback loops, and regulatory processes crucial for sustaining physiological complexity-a hallmark of healthy functioning. Maintaining this complexity enables individuals to effectively adapt to everyday stressors. Conversely, disruptions resulting from excessive rigidity or randomness impair adaptive capacity, highlighting the critical nature of physiological complexity for overall well-being.

We found significant increases in Sample Entropy and MSE at scales 1 and 2 suggesting preliminary evidence of improved autonomic flexibility and adaptive capacity. Elevated entropy values reflect greater complexity in heart rate patterns, suggesting a possible shift toward enhanced autonomic regulation, pending replication [31]. These findings align with previous studies emphasizing that maintaining physiological complexity is indicative of health, whereas its reduction is linked to various pathologies [32,33]. Notably, the intervention appears to have influenced short-term autonomic regulation processes, as indicated by significant entropy changes at lower MSE scales. It is acknowledged that while these changes are statistically significant, their absolute magnitudes are modest, which is expected given that first responders are physically active individuals operating near the upper range of baseline autonomic capacity, limiting the dynamic range available for further complexity improvement. The clinical translation of these preliminary findings requires prospective validation against operationally relevant outcomes in future controlled trials.

The density plots in Figure 2 show Sample Entropy before and after the intervention, revealing a noticeable shift toward higher entropy values post-intervention. This suggests enhanced complexity and reduced regularity in heart rate patterns. The correlation plot in Figure 3 further emphasizes this observation, illustrating a consistent linear relationship between pre- and post-intervention entropy levels, indicating individual consistency in adaptive responses.

The raincloud plots in Figure 4 for MSE scales 1 to 3 highlight significant increases at scales 1 and 2. However, no significant changes were observed beyond scale 3, suggesting that the intervention predominantly influenced short-term regulatory processes. The Bland–Altman plot in Figure 5 illustrates the agreement between pre- and post-measurements of Sample Entropy, with most data points lying within the 95% confidence limits. The shift in the mean difference reinforces the presence of systematic change due to the intervention. Figure 6 illustrates the paired differences in RR and Det, demonstrating reductions post-intervention. These shifts indicate enhanced cardiovascular adaptability and diminished physiological rigidity, key components of improved autonomic flexibility.

Interestingly, no significant differences were observed at higher MSE scales (3 through 20) (Figure 4), suggesting that long-term regulatory patterns may require more prolonged intervention periods to manifest significant changes [34,35]. This finding indicates that immediate improvements in autonomic complexity initially occur in faster-responding systems, while slower adaptive mechanisms necessitate longer durations or increased intervention intensity. Further research exploring extended intervention periods and longitudinal follow-ups is warranted to elucidate deeper autonomic adaptations.

Additionally, the observed reductions in Recurrence Rate (RR) and Determinism (Det) are consistent with the physiological complexity paradigm. This paradigm asserts that healthy biological systems exhibit a dynamic balance between chaotic behavior and coherence, fundamental for adaptive health. Reduced RR values indicate decreased repetitive patterns, suggesting improved cardiovascular responsiveness to stressors critical for adaptive resilience [36]. Similarly, lower Det values reflect less predictable heart rate dynamics, indicating increased flexibility and diminished physiological rigidity, hallmark traits of robust autonomic regulation [37].

Prior research indicates that both excessive regularity and randomness can reduce physiological complexity under stress [38,39,40]. Environmental factors significantly influence HRV, underscoring the importance of personalized lifestyle interventions for cardiovascular health management [41,42].

These preliminary findings are consistent with the ‘physiological complexity’ paradigm introduced by Goldberger and colleagues, which describes healthy biological systems as displaying flexible, multi-scale chaotic dynamics balanced between order and disorder. Loss of this complexity compromises adaptability and physiological functionality [43,44,45]. The physiological mechanisms through which mindfulness interventions plausibly produce the observed improvements in HRV complexity have been discussed in multiple studies. Mindfulness practice is understood to strengthen prefrontal cortical regulation of limbic and hypothalamic stress circuits, particularly the amygdala, attenuating sympathetic hyperactivation and enhancing parasympathetic efference via the vagus nerve, the primary neural substrate of HRV [46,47,48]. Increased vagal tone mitigates the dominance of rigid, sympathetically driven heart rate patterns, thereby generating greater beat-to-beat irregularity and multi-scale complexity reflected in the elevated Sample Entropy and MSE values [49]. Concurrently, mindfulness-induced attenuation of hypothalamic–pituitary–adrenal axis reactivity reduces chronic cortisol exposure, which otherwise suppresses vagal tone and progressively erodes HRV complexity under sustained occupational stress [50,51].

### Why Did Only Nonlinear Metrics Improve?

Traditional HRV metrics such as SDNN, RMSSD, and frequency-domain measures summarize overall variability but may fail to capture the dynamic structure of heart rate fluctuations, especially under stress. Nonlinear metrics, including entropy-based measures (e.g., Sample Entropy, Multiscale Entropy), Poincaré descriptors, and fractal scaling indices, offer deeper insight into the complexity and adaptability of autonomic regulation. These methods have shown greater sensitivity in high-stress and clinical populations. For example, in trauma patients with normal vital signs, reduced Sample Entropy predicted the need for life-saving interventions, whereas SDNN did not [52]. Similarly, studies in tactical personnel revealed that nonlinear measures, but not traditional HRV indices, correlated with fitness and stress [53]. In firefighters, machine learning models identified nonlinear features such as fractal scaling and correlation dimension as top predictors of stress episodes during duty [54]. Furthermore, entropy measures have been shown to reflect physiological adaptability: while sedentary individuals exhibit a loss of HRV complexity under stress, fit individuals maintain or increase entropy, indicating resilient autonomic responses [55]. These previous findings underscore the clinical utility of nonlinear HRV metrics in capturing subtle but meaningful changes in autonomic function, particularly in high-demand environments.

The literature on links between mindfulness and first responder psychological functioning provides additional context for our findings [56]. Preliminary evidence indicates that mindfulness interventions enhance resilience, reducing stress and burnout among first responders by fostering emotional regulation [57,58]. Enhanced resilience subsequently supports improved autonomic regulation and physiological adaptability, aligning with observed increases in HRV complexity metrics [10,11].

## 5. Conclusions

Our findings suggest wearable-based interventions may significantly enhance HRV complexity metrics, indicative of improved autonomic regulation and adaptive capacity among first responders. Although the observed effect sizes are modest, they are physiologically interpretable within a healthy occupational population and represent ecologically valid signals that support further investigation in larger, adequately powered, randomized controlled trials. Future longitudinal studies with follow-up assessments beyond one month are needed to characterize the permanence of these effects and determine the minimum engagement dose required to sustain HRV complexity improvements in first responders. Our results highlight the potential utility of wearable-based stress management programs for enhancing physiological resilience in high-stress populations. Future research should explore the longitudinal effects and underlying mechanisms mediating these autonomic adaptations, particularly regarding the delayed response in higher MSE scales and the stability of entropy changes over time.

## Figures and Tables

**Table 1:** HRV parameters and paired *t*-test analysis for participants (n = 87). Asterisks (*) denote statistically significant *p*-values (*p* < 0.05).

Parameters	Pre-Intervention	Post-Intervention	*p*-Value
Mean HR	79.54 ± 9.80	79.30 ± 9.46	0.14
SDNN	33.50 ± 8.60	33.79 ± 8.29	0.115
RMSSD	31.43 ± 10.16	31.88 ± 9.61	0.311
Stress Index (SI)	9.18 ± 2.28	9.33 ± 2.19	0.156
SNS	0.84 ± 0.99	0.85 ± 0.95	0.11
PNS	−0.98 ± 0.72	−0.95 ± 0.67	0.091
Sample Entropy	1.39 ± 0.10	1.42 ± 0.11	0.007 *
MSE	1.67 ± 0.11	1.69 ± 0.13	0.038 *
Recurrence Rate (RR)	29.21 ± 17.23	28.95 ± 12.20	0.025 *
Determinism (Det)	97.67 ± 30.20	97.59 ± 24.23	0.018 *

## Data Availability

All data collected and analyzed are shared via the FigShare repository at DOI: https://doi.org/10.60911/chapman.29925035.v1 or from the author Dr. Thurmon Lockhart.
